# Biobank resources for future patient care: developments, principles and concepts

**DOI:** 10.1186/2043-9113-1-24

**Published:** 2011-09-16

**Authors:** Ákos Végvári, Charlotte Welinder, Henrik Lindberg, Thomas E Fehniger, György Marko-Varga

**Affiliations:** 1 Clinical Protein Science & Imaging, Biomedical Center, Dept. of Measurement Technology and Industrial Electrical Engineering, Lund University, BMC C13, SE-221 84 Lund, Sweden; 2 Dept. of Oncology, Clinical Sciences, Lund University and Skåne University Hospital, Barngatan 2B, SE-221 85 Lund, Sweden; 3 Institute of Clinical Medicine, Tallinn University of Technology, Akadeemia tee 15, 12618 Tallinn, Estonia; 4 First Department of Surgery, Tokyo Medical University, 6-7-1 Nishishinjiku Shinjiku-ku, Tokyo, 160-0023 Japan

## Abstract

The aim of the overview is to give a perspective of global biobank development is given in a view of positioning biobanking as a key resource for healthcare to identify new potential markers that can be used in patient diagnosis and complement the targeted personalized drug treatment. The fast progression of biobanks around the world is becoming an important resource for society where the patient benefit is in the focus, with a high degree of personal integrity and ethical standard. Biobanks are providing patient benefits by large scale screening studies, generating large database repositories. It is envisioned by all participating stakeholders that the biobank initiatives will become the future gateway to discover new frontiers within life science and patient care. There is a great importance of biobank establishment globally, as biobanks has been identified as a key area for development in order to speed up the discovery and development of new drugs and protein biomarker diagnostics. One of the major objectives in Europe is to establish concerted actions, where biobank networks are being developed in order to combine and have the opportunity to share and build new science and understanding from complex disease biology. These networks are currently building bridges to facilitate the establishments of best practice and standardizations.

## 1. Introduction

The development of gene and protein functional analysis has progressed substantially since the first draft of the human genome was announced a decade ago. These advancements are seen by the increasing number of clinical studies that have been undertaken, and the number of patient samples that have been processed, and investigated by proteomics/genomics-, and bioinformatics studies [1-3]. For example, a search of the term "biomarker" on the United States National Institutes of Health database of registered clinical trials returns 8298 hits http://clinicaltrials.gov/ct2/results?term=biomarkers. This considerable progress in medical science particularly linked to drug development and diagnostics has given us a unique milestone position, from where we have established the new beginning of an understanding of protein function in disease. An estimated $1bn has been invested in the biobanking industry within the last ten years. At least 179 biobanks with 345,000 donors exist in the US, most of which were established in the last 10 years (source: Business Insights, March 2009).

The genetic link to disease has been very closely aligned to the bioinformatics disciplines and the building of databases and software search engines. This was recently exemplified by Venter in his groups first description of the idea of creating an artificial genome with specific functions [4]. This vision came from sequencing hundreds of marine microorganisms and forms the basis of a giant database containing protein-coding sequences from hundreds of microbial genomes therein http://www.jcvi.org/. These futuristic developments are expected to become a great value to mankind as we relate specific proteins to pathways associated with disease.

Understanding the mechanisms by which specific protein functions contribute to disease pathogenesis is a great challenge. In comparison to the genomic map, the proteome map might be 100 times larger. Studies with model organisms such as *Drosophila melanogaster*, *Saccharomyces cerevisiae *and in man have aligned specific protein functions to pathways as node structures both at the level of intracellular organelles but also in whole organisms in protein-protein interaction maps [5-7]. Further linkages have bee made to cluster genes associated with one or another of the 1500 described medical disorders in what has been named the human diseaseome [8]. These associations will form the basis for producing models of inheritance, exposure, and possible clinical outcomes linked to gene expression and subsequent protein functions.

Proteins are, unlike the human genome, dynamic targets that constantly change not only their relative abundance levels but also their physical forms. This is one important reason why the protein area has a much higher complexity and more variable in human populations. In this respect, the resting steady state of a protein, may change its form and function during a disease development such that the activation state of a protein is perturbed by in most situations the post-translational modifications of the gene encoded protein sequence by for example phosphorylation, glycosylation, oxidations, alkylations and acylations.

Since protein structures and protein functions are the most common targets of drug therapy there is great interest to develop new paradigms of therapy based upon antagonist or agonist drivers of specifically targeted proteins. Drug development meeting this challenge is prone for difficulty in avoiding off target interactions due to our inability to predict all possible interactions with any given drug with all proteins in the human proteome. One can imagine that differing drug-protein interactions occurring at differing concentrations of the active substances, their relative retention times in tissue, and their metabolism to inactive forms.

These difficulties are reflected in the small number of new medical entities introduced annually as new agents into the marketplace. For novel drugs with improved efficacy properties, it is important to optimize the affinity interaction in-between the protein target and drug molecule, with a large safety window (dose-response characteristics), and minimal off target effects or toxicity. Lately, the patient safety assessments have been the major focus for FDA, requesting additional extensive and large-scale clinical trials, in order to provide statistical significance on new drug properties.

**Figure 1:**
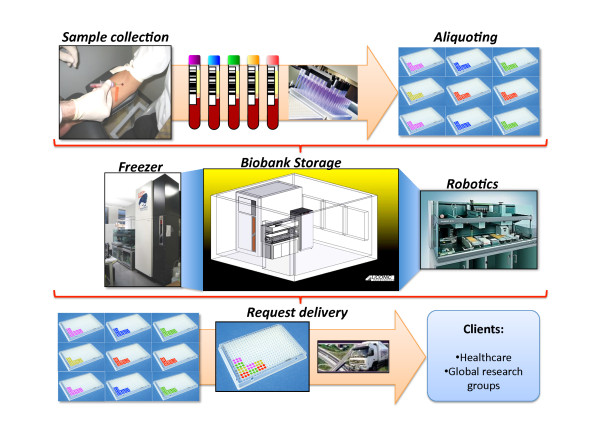
**Biobank structure with its links to the health care area**.

Large international consortium and research initiatives are common in modern medical research that utilizes clinical biobank samples. International standards are being developed and implemented which will make large global comparative studies possible [9,10]. The biomolecules that are currently of major value in modern biobanking, retained in biofluids and tissues are DNA, mRNA, proteins, peptides, phospholipids, and small metabolites. DNA is a very stable molecule, and can be isolated from patients. The protocols applied for DNA vary in global biobanks, but would not be expected to impact on the quality of the analysis data generated. Proteins and mRNA, degrade to a varying extent in bio-fluids, and thus present a major challenge for biobank establishments. Sampling, sample preparation and sample processing protocols are of principal importance to preserve the quality of the final stored samples. This is also true for fatty acids and metabolites, in clinical samples that represent future potential biomarkers. The workflow of the various part of the biobanking process is outlined in Figure 1.

Not too long ago, in the 90's it was widely believed that the human proteome contained around 2000 proteins. From the Human Genome Initiative, today we are aware of the approximate number of 20,300 human proteins, encoded by the genome. These estimates were based on statistical links that were established at the time, between peptide mass fragment spectra in existing databases and amino acid sequences predicted from the genomic databases. But the actual number of unique protein forms in the proteome is estimated to be much higher. Taking into consideration gene allelic expression variations and mutations, spliced variants of mRNA species, and differing types of post translational modifications both within and outside the cell, we can already estimate that hundreds of thousands of different protein form may be expressed during a lifetime. With the splice variants and posttranslational modifications, the number will reach many million proteins within the human body.

Interestingly, there are limited controls of the quality of samples that are collected globally in large archives. There also seems to be a shortcoming of assays, and standardized systems whereby the degradation levels of biomolecules in a given biofluid present in biobanks can be controlled. In addition, diagnostic platforms and assays that can verify the disease stage and progression is only applied for biobank sample characterization to a limited extent.

In fact, it is also fair to state that a lot of promises and Wall Street expectations on biomarkers have yet to be manifested [11]. The technology driven disease biology cataloging exercise is a greater challenge than expected. Another great endeavor has been started and initiated: The Human Proteome Project (HPP) that was launched in September 2010 in Sydney at the HUPO World Congress [9]. This idea and science project outline was already presented by Anderson and Anderson several decades ago [12].

So far 10 global chromosomal consortia has been initiated with the objective to sequence all proteins of a given chromosome, coded by the genome [13-15]. One of the several goals of this global initiative is to utilize well-characterized clinical material from biobanks where patients have been given their dedicated contribution to human wellness by development of personalized medicine and dedicated diagnostics. The Chromosome 19 Consortium will be collaborating with a number of biobanks and clinical hospitals around the world.

**Figure 2:**
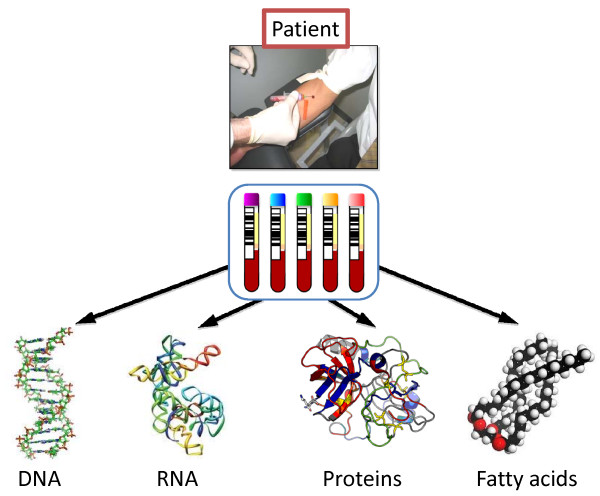
**Illustration of the analytical technologies targeting the broadest range of biomolecules utilizing biobanking materials**.

All of these developments and progresses in modern biomedical research have now been identified as a starting point for the establishment of large and well-characterized modern biobanks. These biobank units, collected and archived on a national level, are being developed with the common goal for optimizing the storage of samples and developing high-end analyses platforms for measuring markers present in clinical samples for research and development purposes (Figure 2). Health care institutions as well as research teams merge and meet within the establishments of Biobank institutions, where the collective sample sets of today will become the tools for diagnosing and monitoring disease development and responses to therapy in the future.

It is also evident that the substantial advancement of research on the human genome and protein science has led to the creation of biobanks, that have brought forward a paradigm shift in drug testing and development. Recognizing the potential benefits from biobanks, pharma and biotech across the world are investing in infrastructure and biobank development. The pharmaceutical industry is currently establishing collaborative efforts with principle investigators (PI), within hospitals, or the academic medical area. Secondary biobanks are also established where the primary biobank, *i.e*., the hospital will provide sample sets from the study. In these projects, pharma companies will be handling the shipments from the hospital, and will provide adequate administrative and freezer capacity for storage and analysis [16].

## 2. The importance of biomarkers for target identification and validation

In many instances the role of a protein is not so straightforward with respect to its disease function. The protein can act as a drug target, but in many instances also as a biomarker. The ultimate role of a protein is to verify its role and function in a given disease pathology, understanding the progressive disease mechanisms.

The utilization and development of novel diagnostic biomarkers have a great potential, where both industry and the academic field are investing and exploring approaches to tie together technologies to make innovative discoveries. There are currently many putative diagnostic biomarkers to be assessed. However, these candidates will need validations in clinical studies, to determine which combination of markers has the greatest diagnostic and prognostic power. In addition, biomarkers are playing a key role in drug development. In fact, diagnostic biomarkers are also of mandatory importance in selecting the patient group for a targeted personalized treatment as well as for safety considerations.

In fact, assays for diagnostic application of protein analysis is a priority and is increasing. Advancing protein analysis for clinical use, is aimed towards diagnostics and biomarkers, where proteins exists and have been used as markers of disease for more than 150 years [17].

Today, biomarkers are being assessed in clinical drug studies, where three categories of markers usually are assigned; biomarkers as proof of principle, biomarkers as proof of mechanism and, biomarkers as proof of concept [18]. Decision on progress of the drugs in clinical study phases is made from the resulting outcomes of these biomarker assays.

## 3. Biobank resources

Health care organizations worldwide strive to seek the best cure for patients, suffering from various diseases. The healthy population in relation to patients forms the basis for biobank strategies where the search for an understanding of diseases at a molecular level is at focus. The aim of collecting samples from patients is to try to discover common patterns and molecular signatures of disease and disease stages. Most developments in the area are aimed towards the discovery, and understanding diagnosis implementations, providing the right treatment alternatives for patients.

The challenges for providing accurate markers of disease are increasing, and related to problems that are due to the multi-factorial disease indications that nowadays can be identified by modern imaging technologies and molecular diagnosis. In most cases, it is impossible to align a given disease diagnosis to a single molecule that is uniquely related to one disease, or clinical complaint. On the contrary, there are typically hundreds of such biological signal read-outs (high density array signals), in modern biomarker diagnosis, which may complicate the identification and selection of the important factors that can work as indicators of disease.

The quality of human clinical samples, such as blood fractions, tissues, that can be both freshly frozen, as well as paraffin embedded and formalin fixed is in the center of most disease studies. The analysis technology platforms will be directed towards DNA, RNA, proteins and metabolites. In these assays, antibody based assays, as well as gene clone collections, siRNA libraries, affinity binders, primary cells, and the development, or use of existing cell-lines.

## 4. Investments into society

The social welfare systems, that deliver medical care, are today in a state of major restructuring and change. In order to meet the limitations in everyday health care, that is lacking both resources, as well as targeted treatment efficiency, changes are needed. High quality treatments in most common diseases, such as cancer, cardiovascular diseases, neurodegenerative diseases, and diabetes, to mention the most resource demanding, is something that the patients are desperate about. This is certainly a global trend and development, rather than local needs. The health care sector is in great need of improvements in efficiency on all levels. This is a valid statement for most countries in the world. Consequently, a legitimate consideration would be to ask the question: For what purpose are Governments and Private Foundations ready to invest into this research field? The main strategy in developing biobank resources across the world is to be able to improve on the prevention, diagnosis, as well as treatment of disease and to promote the health of the society [19]. Considering biobank resources as an added value to build the future health care, some positioning in society and clarification requirements arises. These relate for instance to: *"What does biobanking mean?"*

A common reflection that is given by persons on the street with no experience or specialist background. Biobanks are by no mean a new concept, or idea. Blood banks have been an integral part of medical care for more than 100 years. The science of sampling and storing whole blood and blood products has made great advancements not the least of which are the registers of healthy volunteers that provide the samples and maintain the resource. For research purposes, in Scandinavia, doctors in hospitals have also been collecting samples for more than a hundred years. The aim of these studies has been to get a better understanding of the presentation of disease within patient groups and how best to understand the correlation to clinical measurements. Today, biobank is a clinical area undergoing a fast and progressive development. It is clear from public legislation and investment the establishment of biobanks around the world has become an integrated part of modern healthcare [20]. In many countries biobanking is organized as a core facility within the hospital clinical chemistry structure, with links to pathology and diagnostic activities. In other nations, the biobank has become an autonomous part of the healthcare industry [21]. The biobank concept is in a phase of development where the implementation into the clinical organization is ongoing, with a varying degree of integration, in Europe, North America and Asia.

In relation to these concepts, each society is expected to be able to offer improved prognosis, at a reduced cost to the healthcare system by early disease indication, with personalized treatment and evaluation of responses to treatments.

## 5. Biobanks, ethics, and personal integrity

The whole Biobank area is going through a major re-building phase where law and regulations are scrutinizing the structure, organization and sample tracking process much more than was commonly practiced in the past. There are important considerations for the protection of individual privacy and personal integrity that must become a focus of any discussion on the collection of individual samples into biobanks. First and foremost is the issuance of informed consent from the patient or study subjects for the inclusion of their specific samples within the biobank. In many countries this is controlled by law and overseen by regulators in local or national governmental bodies. It is often required that informed consent be provided in written format, whereby the intended use of the sample is clearly provided, as well as the means for withdrawing such permissions for future use. Secondly, the commercial exploitation of these sample banks is also much more tightly controlled. These measures provide the individual and society a set of basic rights and entitlements as to the use of their clinical samples in research and or commercial tissue banks. Two such examples of national legislation that provide the ethical and structural basis of obtaining samples for use in biobanks are The Human Tissue Act (2004) in Great Britain and the Biobank Law of Sweden (2002:297). Further examples of documents outlining the infrastructures of sample collecting and sample use can be found in the accompanying references [22,23].

## 6. Patient benefit from biobanking

The study of health and disease in nation wide populations is an important global endeavor that demands large-scale source of investment into infrastructure, surveillance programs, and education and training activities within various levels of the general public. The rising costs of health care could be partially addressed by systems that allowed clinical data to be collected and addressed centrally by health care providers irrespective of the location of the data acquisition.

On a European level, Biobanking and Biomolecular Resources Research Infrastructure (BBMRI) is a European Union initiative from Brussels that involves more that 200 organizations in 24 EU Member States are jointly planning a EU infrastructure http://www.bbmri.eu. The BBMRI vision is that BBMRI sustainably will secure access to biological resources required for health-related research and development intended to improve the prevention, diagnosis and treatment of disease and to promote the health of the citizens of Europe.

In Scandinavia, and with Sweden and Denmark as examples, there has been a long tradition of longitudinal epidemiological studies within the general population. For instance, The Swedish Twin Registry started in 1960 is the largest such registry in the world with currently over 86,000 twin pairs under current study [24,25]. Denmark has a similar registry of Twins [26]. Along with the sample collections, clinical data and information from the participants in the study are collected in national registers. Other Swedish national population registries have studied the health status and collected samples of men evaluated at age 50, born at decade intervals since 1913 (1913, 1923, 1933, *etc*.) [27,28]. Further registries kept primarily at Statistics Sweden as well as the National Board of Health and Welfare include: i) the Hospital Discharge Registry, all diagnoses and medical treatments since 1961; ii) the Cancer Registry, are all collected cases of cancer since 1958, which can be related to the cause of Death Registry, and all underlying causes which is an important asset. It is also possible to follow and provide data that relates to the medical history of patients along with the medical Birth Registry. These extents of these medical resources are probably in the absolute frontline of international standards. The ability to align large data registers with everyday treatments of patients is absolutely necessary and is expected to grow considerably in the near future. The benefit to patients will be the utility to align biobank sample output, to pathological findings and correlations that can aid in modern disease treatments.

## 7. Building qualitative biobank resources

There are many decisions that need to be taken when a biobank facility is to be built and installed. The very first thing that comes to mind is the qualitative aspect of sampling the patient samples and processes them according to a standard operating procedure (SOP). This part is of great importance in order to make the samples comparable in studies that are to follow with the archived material. The sample volumes that need to be stored along with the density of sample racks into where the patient samples are aliquoted will determine the capacity of the biobank freezer needed for storage. The statistical number of samples that is generated will in most cases determine the degree of automation that will be needed in the biobank.

These are strategic decisions that need to be made on the tasks presented above. There will be practical limitations where the number of samples and aliquots will guide towards a route for automated handling. There are exceptions, like the Framingham heart center biobank facility http://www.hcmw.com/, where most of the sample handling is performed manually.

Currently, there are no international qualitative requirements with respect to the samples. Ongoing standardization studies, developments and networking will result in a globally accepted quality aspect of biobank samples and processes.

## 8. Data repositories

**Figure 3:**
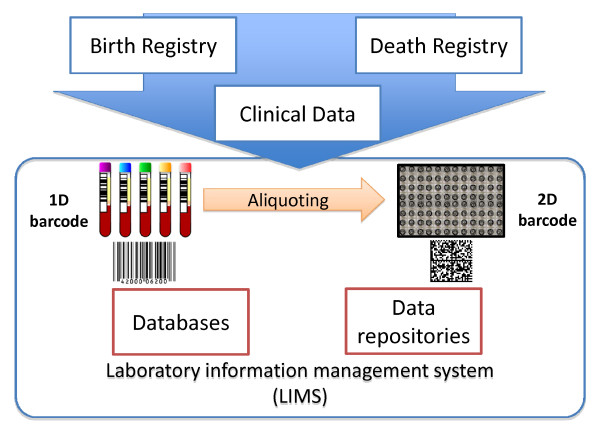
**1D bar code and 2D barcode system, Databases, data repository and laboratory intelligent management systems**.

The barcode is the common nominator and identifier of a sample. This code can be utilized in both 1D and 2D form, capturing important identifiers for each sample type and origin. The bar-coded information is aligned to the clinical data and details from the data registers (as presented above). The laboratory information management system (LIMS) is the software interface that stores and manages all data associated with the sample including it's history, storage location and storage lifetime as well as linking to additional databases of clinical measurement data associated with the subject (see Figure 3). The LIMS also provides data on the history of each sample tube use that is fully traceable. There is a also an imperative need to be able to follow and track down the sample history of any given donation given by patients in clinical studies, in the case that study subject requests to be excluded from the sample repository.

Data repository systems are built within mega-sized databases where this "intellectual center" can be reached and interfaced, in principle from any global location.

Biobanks in the world that have been in operation for decades with extensive experience and track records, such as the Framingham heart center http://www.hcmw.com, the UK Biobank http://www.ukbiobank.ac.uk and the Singapore Bio-Bank, a research tissue and DNA bank http://www.stn.org.sg. We can already forecast that these forms of sample repository could face potential challenges in the future regarding specific requirement for sample handling posed by future studies. For instance not all stored disease specific and/or population-based sample collections will be able to meet the future demand for criteria such as frozen samples without thawing history. If samples are stored in larger sample volumes, it is often the practice to thaw a complete sample volume in order to obtain a fraction for analysis. Over the years of testing, such samples could be aliquoted many times with intervals of freezing and thawing. This is today not the preferred strategy. Instead, aliquoting of small sample volumes and higher aliquot numbers is the preference.

No doubt, there are major biobank stakeholders in this new field, where major investments are currently being made. We are awaiting novel solutions of future biomarker deliverables, such as preventive-, and drug-targeted biomarkers, as well as new imaging diagnostic technologies. These new conceptual developments are especially urgent due to a high unmet need within diseases such as cancers, obesity, diabetes, cardiovascular diseases, and others. Introducing biobanks as a new powerful modality within the field of modern life science is expected to be important in promoting pro-active awareness of patient health status. The pro-active concept should be seen as a future investment for many countries. The current strategy will build future capacities, instead of the act-on-demand practice that is often undertaken, when the patient already has reached more advanced disease stages. Such, so-called preventative medicine activities are already being implemented in Japan as a standard health care activity. The result is to reduce hospital admissions by diagnosing and treating early and thus save the high cost of extended hospital care required with advanced disease. Biobanking may play a key role in this process by providing standards for biomarker measurement in the form of personalized indicator assays that could be coupled to individual treatment schemes [29].

Large biobank facilities equipped with robotics and automated sample processing will also become an important asset for pharmaceutical drug development. The development of new more effective drug therapies is neither easy nor straightforward. The targets of these drugs, often proteins, need to be understood and this understanding only comes from studying expression in various disease states. Biobanks of diseased and non-diseased subjects can provide the differential measurement of the change in expression that occurs during disease transition.

In addition, each biofluid and/or tissue sample will most probably have associated clinical data, from where the patient cohorts can be composed. It is also envisioned that the biobanking initiatives will generate a whole new set of data sets from expression studies. These new data sets will be a valuable delivery, and payback for accessing the treasures within biobanks. Large protein expression studies, using LC-MS, have been undertaken, where differential quantitation of proteins, present in healthy and diseased patient groups, has been identified. The bio-statistical analysis outcome and bioinformatics leverage of disease studies, where drug effects, and drug safety, are the objectives, will have an increased impact if medical informatics are assigned to these data. The combination of bioinformatics results that are aligned with clinical measurements, and medical history data will stand a better chance in picking up correlations where disease specificity can be directed to a given patient phenotype [30].

It is with great interest that we will follow the maturation of mechanistic disease pathophysiology, based upon gene and protein expression. The HUPO Chromosome Consortia in collaborative efforts with the proteomics society will build the future basis of the human proteome. The deliveries will be publicly processed and available in several of the public data repositories, such as UniProt, PRIDE and Tranche [31-35].

Another objective, that needs to be met, will be the protein data integration, with functional networks that will provide us with a comprehensive data set, to be used as a public resource.

## 9. Screening technology platforms

There are a number of technology platforms that are readily available for sample characterization, that is helpful in cataloging the biobank content, and what is available for experimental access. Traditionally, protein-based clinical chemistry assays have played a major role in health care treatment and diagnosis of patients. In many countries around the world, about 109 protein markers are in use for medical treatments [17]. The initiation of the Human Proteome Project (HPP), where the chromosomes are being sequenced with respect to gene coding regions resulting in protein synthesis, is expected to increase the availability of both drug target studies as well as pathology, and biomarker investigations [36]. As we are celebrating the decade anniversary of the human genome, consequently, gene expression profiling and new generation sequencing, that allows high speed and turnover data generations in a format that previously has been impossible, also opens up for biobanking outputs [37-39].

NMR spectroscopy is a technology platform used for metabonomic analysis in order to discover new biomarkers as well as to track down metabolite information, implicating definite putative protein targets in a given toxicological mechanism. Typically blood plasma, urine and liver samples are being screened in these studies and resultant spectra are being correlated to sequential 1H NMR measurements with using pattern recognition methodologies [40-42].

In our group we are investigating the opportunities in building high content biobanks. In these developments, we are linking the corresponding clinical data that can be assigned to each little fraction of a patient sample in the sample repository. We recently reported on the development of a stable isotope-labeled peptide strategy, to control sample stabilities within biobanking [43].

Reference standards can be used by their qualitative and quantitative changes, using MALDI MS and nanoLC-ESI MS. We have shown a concept where we are able to follow the degradation process in human blood plasma samples by monitoring the changes of these three peptides [43].

In addition to this sample characterization, we use disease staging and pathological grading, as well as clinical assay screening as standard procedure.

### 9.1 Multiple Reaction Monitoring (MRM) Assays

Biobanking developments provide large amount of clinical samples available for analysis of protein biomarkers, which are recognized as differentially expressed in comparing clinical status of disease and health. Mass spectrometry (MS) is currently the most frequently applied sequencing-, and detection platform when interfaced to liquid chromatography (LC). Both targeted as well as non-targeted LC-MS profiling technologies, are being applied to protein, peptide, and metabolite profiling and differential expression analysis [18].

Studies are conducted by global expression analysis, where a non-directed principle is applied, where many thousands of proteins and/or small molecules can be analyzed and sequenced in a small amount of sample. Studies where the analytes of interest are known, is measured by a targeted approach, where a specific and smaller set of analytes are measured in dedicated assays. In the last years, MRM multiplex assay have become very popular due to their generic concept [44,45].

Following biomarker validations, MRM offers quantifications of proteins in complex biological matrices measuring peptide levels [46]. In combination with appropriate stable isotope-labeled internal standards, the MRM approach provides absolute quantitation of the analyte [47]. Additionally, a high number of proteins of interest can be monitored simultaneously in MRM assays [48].

**Figure 4:**
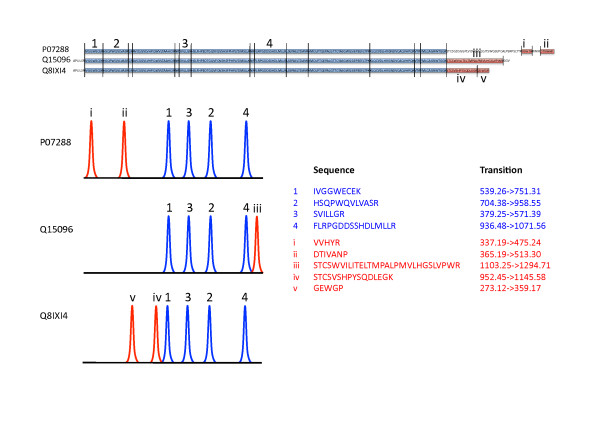
**Comparative quantitation of three PSA isoforms (access codes: P017288, Q15096 and Q8IXI4) by MRM assay**. Blue and red parts of the sequences represent identical and isoform specific tryptic peptides, respectively.

The MRM quantifications present high sensitivity and speed, which is a future requirement for high throughput screening of clinical samples for candidate biomarkers within the clinical study area. Currently, MRM applications are the fastest growing targeted protein analysis area, with multiplex assays for absolute quantitation in clinical disease areas. For these reasons, we utilize the MRM technology in quantitation of prostate specific antigen (PSA) isoforms in clinical samples (Figure 4). PSA is the only biomarker used for diagnosis of prostate cancer in many countries as a routine clinical measure. Increased levels of PSA indicate a potential problem of early onset stages of prostate cancer. The number of ELISA test kits used in everyday diagnosis [49] may not recognize several molecular forms of PSA as we have recently shown (Végvári Á, Rezeli M, Sihlbom C, Häkkinen J, Carlsohn E, Malm J, Lilja H, Laurell T, Marko-Varga G: Molecular Microheterogeneity of Prostate Specific Antigen in Seminal Fluid by Mass Spectrometry. *Clin Biochem*, 2011) [50]. The addition of quantitative information to these newly identified molecular forms of PSA may eventually lead us to improved diagnosis of prostate cancer.

### 9.1 Flow Cytometry

**Figure 5:**
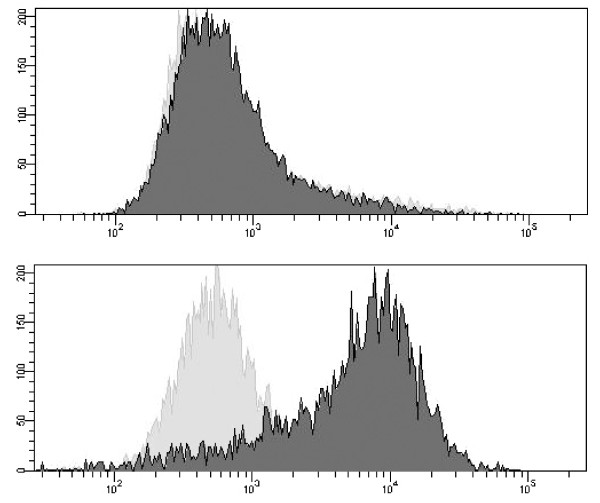
**Analysis of a surface marker on two different cell lines by flow cytometry**. Histograms showing unlabelled control cells (solid gray area) and fluorescently labeled cells with a surface marker (solid black area). A) showing a clear positive expression and B) no expression of the surface marker.

Flow cytometry is another technology platform whereby biobank samples can be characterized. The technique is powerful and provides rapid analysis of multiple characteristics of single cells and is both qualitative and quantitative. In flow cytometry individual cells are held in a stream fluid and the cells are passed through one or several laser beams, which cause light to scatter and fluorescent dyes to emit light at various wavelengths. The forward scatter measures cell size, while the side scatter determines the complexity within the cell. Using fluorescent labeled antibodies in combination with flow cytometry can reveal the presence of specific proteins on the cell membrane or inside the cell (Figure 5). A variety of samples from biobank can be used *e.g*., whole blood, bone marrow, cerebrospinal fluid, urine and solid tissue.

**Figure 6:**
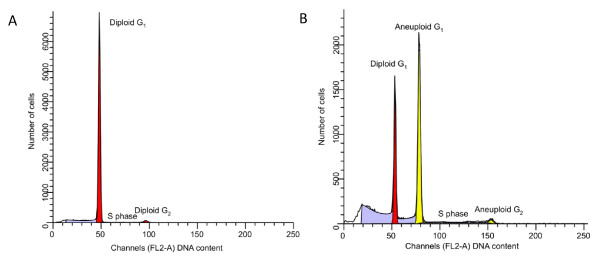
**Comparison of histograms**. (A) Histogram from an ovarian diploid cancer: Red population: Flow cytometric DNA index: 1.00, S phase fraction: 1.5%. (B) Histogram from an ovarian non-diploid cancer: Yellow population: Flow cytometric DNA index: 1.47, S phase fraction: 11.9%. Red population corresponds to the contribution of DNA diploid (DNA index: 1.00) cells in the tissue sample.

Today, flow cytometry is used in clinical laboratories for applications, such as DNA content analysis (ploidy) and proliferation analysis (S-phase) as shown in Figure 6. In different tumor tissue both aneuploidy and a high S-phase have been correlated to a poorer prognosis for the patient. Flow cytometry is also used for leukemia and lymphoma phenotyping, immunologic monitoring of HIV-infected individuals.

## 10. Conclusions

How large of a role that Biobanks will play in the development of new paradigms of disease pathogenesis and in the establishment of new treatment protocols for unmet needs in the clinic will only be learned in time. If the answer can be found in stored samples, representing milestones of health and illness, this deserves attention by the public and the political institutions that protect the public's interest. Lastly, whether such future solutions will be able to provide the remedy and become the Holy Grail of disease understanding, still remains to be proven by all of us within the scientific and industrial community.

Automation and unattended robotic processing of biobank samples are current an area of great expansion and development where many research groups and instrumental companies are very active. Still, its fair to say that some biobanks, even well reputed as the Framingham heart center, uses manual handling of patient samples. This is on the other hand an exception.

The automation is wide spread when it comes to liquid handling and sample aliquoting. Here we have liquid handling robotics of various sizes and capacities that can manage even complicated aliquoting and processing. The sample handling within -80°C and robotic storage is another matter where currently many teams and companies are developing large capacity units that can store many million of patient samples.

The size and density of the rack holders, and how many tubes that can be fitted into a 12 × 8 cm area is still a challenge that we will see systems built from in a very near future.

## 11. List of abbreviations used

BBMRI: Biobanking and Biomolecular Resources Research Infrastructure; CTC: Circulating tumor cells; FDA: Food and Drug Administration; HUPO: Human Proteome Organization; HPP: Human Proteome Project; LIMS: Laboratory information management system; PI: Principle investigator; SOP: Standard operating procedure; MRM: Multiple reaction monitoring; MS: Mass spectrometry; LC: Liquid chromatography.

## 12. Competing interests

The authors declare that they have no competing interests.

## 13. Authors' contributions

The authors contributed equally to this work. All authors read and approved the final manuscript.
